# Patterns of malaria-related hospital admissions and mortality among Malawian children: an example of spatial modelling of hospital register data

**DOI:** 10.1186/1475-2875-5-93

**Published:** 2006-10-26

**Authors:** Lawrence N Kazembe, Immo Kleinschmidt, Brian L Sharp

**Affiliations:** 1 Applied Statistics and Epidemiology Research Unit, Mathematical Sciences Department, Chancellor College, University of Malawi, Zomba, Malawi; 2 Malaria Research Programme, Medical Research Council, Durban, South Africa

## Abstract

**Background:**

Malaria is a leading cause of hospitalization and in-hospital mortality among children in Africa, yet, few studies have described the spatial distribution of the two outcomes. Here spatial regression models were applied, aimed at quantifying spatial variation and risk factors associated with malaria hospitalization and in-hospital mortality.

**Methods:**

Paediatric ward register data from Zomba district, Malawi, between 2002 and 2003 were used, as a case study. Two spatial models were developed. The first was a Poisson model applied to analyse hospitalization and minimum mortality rates, with age and sex as covariates. The second was a logistic model applied to individual level data to analyse case-fatality rate, adjusting for individual covariates.

**Results and conclusion:**

Rates of malaria hospitalization and in-hospital mortality decreased with age. Case fatality rate was associated with distance, age, wet season and increased if the patient was referred to the hospital. Furthermore, death rate was high on first day, followed by relatively low rate as length of hospital stay increased. Both outcomes showed substantial spatial heterogeneity, which may be attributed to the varying determinants of malaria risk, health services availability and accessibility, and health seeking behaviour. The increased risk of mortality of children referred from primary health facilities may imply inadequate care being available at the referring facility, or the referring facility are referring the more severe cases which are expected to have a higher case fatality rate. Improved prognosis as the length of hospital stay increased suggest that appropriate care when available can save lives. Reducing malaria burden may require integrated strategies encompassing availability of adequate care at primary facilities, introducing home or community case management as well as encouraging early referral, and reinforcing interventions to interrupt malaria transmission.

## Background

Measuring malaria burden in a population is a challenge in most developing countries [1,2]. Routine hospital data, reported through the health management information system (HMIS), provide a proxy for measuring the incidence of severe malaria and for crudely measuring morbidity rates [3,4]. Describing trends and patterns of such data could assist in monitoring and planning resource needs in a health system [5]. In particular, explaining geographical variation in such outcomes is important to identify communities at high risk, to assist in designing appropriate interventions, or lead to further investigations to identify important risk factors.

Use of spatial analysis is increasingly being applied in epidemiological research in recent years [6], and examples of applications in malaria are expanding [5,7,8]. Availability of geo-referenced health data, advances in statistical methodology and developments in geographical information system (GIS) are the reasons for such increased trends [6]. Despite growing applications of spatial methodology in malaria research, and regardless of several studies using hospital data to explore the burden of malaria [9-12], fewer studies have analysed spatial variation of hospitalization and in-hospital mortality rates [5,13].

Because malaria transmission intensity varies geographically, the distribution of hospital cases and subsequent mortality rates may exhibit systematic spatial variation [14]. Thus spatial analysis may shed light on geographical variations in hospitalization and mortality rates. The main objective of this study was to apply a spatial modelling framework, as a case study, to describe malaria hospitalization and in-hospital deaths using paediatric hospital data from Zomba district in southern Malawi. Two types of spatial models were used. In the first analysis, Poisson models were fitted to analyse rates of hospitalization and in-hospital mortality, adjusted for age and sex, which can be interpreted as population-based cohort studies. In the second, individual-based observations were used and a logistic regression model was applied to assess individual covariates influencing in-hospital mortality.

## Methods

### Study area and data source

Zomba, one of the 28 districts located in southern Malawi, is generally classified as malaria endemic, although the highland zones in the central parts of the district are of low transmission and may be prone to epidemic malaria [15-17]. In 2002, the district contributed about 4% to the national total of in-patient malaria cases [18].

Data used in this study were obtained from discharge records of all paediatric hospital admissions at Zomba district hospital, Malawi, between 1 January 2002 to 31 December 2003. Zomba district hospital, with over 500 beds is the largest facility in the district which serves both as the first consultation point for patients within its catchment, and as a referral centre for other 25 primary health centres. These facilities are managed by the Ministry of Health and the Christian association of Malawi.

For this study, cases with primary diagnosis as malaria, from the hospital wards, were used. Each cases was clinically assessed and definitively confirmed as malaria on admission. The registers included patients' age, sex, date of admission and discharge, outcome (i.e. death, discharged home, home-based care or absconded), village or location of residence, and treatment given. Based on the name of the village, each case was matched to one of 21 residential wards in the district. Approximately 86% of cases were successfully linked to wards, the other 14% having either missing or insufficient residential information. Only geo-referenced cases were included in the spatial analysis, using ward as the spatial unit. Population at risk data for the age (< 1, 1–4, 5–9, 10–14 years) – sex – residential ward strata, projected from the 1998 census, were obtained from the Malawi National Statistics Office [19].

From the data, the following the variable were derived: *day *when admitted (1 = weekend; 0 = weekday); *season *of the year when admitted (1 = wet season from October to March, 0 = dry season from April to September); *treatment *given (1 = quinine, 0 = other antimalarial drugs); *distance *to the hospital (1 = distance ≤ 5 km, 0 = distance > 5 km). The distance of 5 km was chosen to reflect travel time of 1 hour [20,21]. Length of hospital stay was divided into five categories: 1 day, 2 days, 3 days, 4–6 days and 7–20 days. In addition, the variable *referral *was defined, with children who used the hospital as a first point of consultation given a code 0 and those referred to the district hospital from peripheral health facilities in the district given the code 1.

### Statistical analysis

#### Population based analysis

Based on the number of hospital admissions or mortality and the population at risk in each age-sex-residential ward stratum we fitted a Poisson model. More specifically, let *y*_*ijk *_be the total number of cases observed in age group *k *(*k *= 1, 2, 3, 4), sex *j *(*j *= 1, 2) and residential ward *i *(*i *= 1,..., 21), and *N*_*ijk *_the corresponding population at risk. The *y*_*ijk *_are Poisson distributed with expectation *μ*_*ijk*_, given all the random effects. The Poisson regression model for hospitalization and minimum mortality rates has the form,

log (*μ*_*ijk*_) = log (*N*_*ijk*_) + *β*_0 _+ *β*_1 _*Age*_*k *_+ *β*_2 _*Sex*_*j *_+ *s*_*i *_+ *u*_*i*_,     (1)

where log (*N*_*ijk*_) is an offset, *β*_0 _is an intercept, *β*_1 _and *β*_2 _are regression coefficients corresponding to age and sex covariates respectively. The terms *s*_*i *_and *u*_*i *_are random effects that allow for spatially structured variation and unstructured heterogeneity respectively.

#### Individual based analysis

Defining *y*_*ij *_= 1 if the child died in hospital and *y*_*ij *_= 0 otherwise, *y*_*ij *_is a Bernoulli outcome with expected probability of dying equal to *p*_*ij*_, which is interpreted as the *case fatality rate *(CFR). The CFR is modelled using the logistic regression model, i.e.,

logit (*p*_*ij*_) = *η*_*ij *_= x′ij
 MathType@MTEF@5@5@+=feaafiart1ev1aaatCvAUfKttLearuWrP9MDH5MBPbIqV92AaeXatLxBI9gBaebbnrfifHhDYfgasaacH8akY=wiFfYdH8Gipec8Eeeu0xXdbba9frFj0=OqFfea0dXdd9vqai=hGuQ8kuc9pgc9s8qqaq=dirpe0xb9q8qiLsFr0=vr0=vr0dc8meaabaqaciaacaGaaeqabaqabeGadaaakeaacuWG4baEgaqbamaaBaaaleaacqWGPbqAcqWGQbGAaeqaaaaa@3115@*β *+ *s*_*i *_+ *u*_*i *_+ *f *(*t*)     (2)

where *η*_*ij *_is an additive predictor for the *j*th child of residential ward *i*, *β *is a vector of unknown fixed regression parameters, corresponding to the set of covariates (*x*_*ij*_), and *s*_*i *_and *u*_*i *_are the random effects for the *i*-th ward of residence as defined above. The calendar effect, at month *t*, is included through *f *(*t*). Bivariate logistic models were fitted for each covariate (Table 3), and only significant covariates were included in the multiple spatial logistic model.

#### Model inference

For the non-spatial bivariate logistic models a maximum likelihood approach was applied to estimate model coefficients. For the spatial models (Equation 1 and 2), a Bayesian approach was used for inference. The following prior distributions were specified for the parameters in Equations 1 and 2. A conditional autoregressive (CAR) prior [22], was chosen to model the spatial correlation effects. This assumes that contiguous areas have similar risk patterns. The CAR prior has the form

p(si|sl;τs2)∼N(1mi∑l≠isl,τs2mi)     (3)
 MathType@MTEF@5@5@+=feaafiart1ev1aaatCvAUfKttLearuWrP9MDH5MBPbIqV92AaeXatLxBI9gBaebbnrfifHhDYfgasaacH8akY=wiFfYdH8Gipec8Eeeu0xXdbba9frFj0=OqFfea0dXdd9vqai=hGuQ8kuc9pgc9s8qqaq=dirpe0xb9q8qiLsFr0=vr0=vr0dc8meaabaqaciaacaGaaeqabaqabeGadaaakeaacqWGWbaCcqGGOaakcqWGZbWCdaWgaaWcbaGaemyAaKgabeaakiabcYha8jabdohaZnaaBaaaleaacqWGSbaBaeqaaOGaei4oaSdcciGae8hXdq3aa0baaSqaaiabdohaZbqaaiabikdaYaaakiabcMcaPiablYJi6iabd6eaonaabmaabaWaaSaaaeaacqaIXaqmaeaacqWGTbqBdaWgaaWcbaGaemyAaKgabeaaaaGcdaaeqbqaaiabdohaZnaaBaaaleaacqWGSbaBaeqaaaqaaiabdYgaSjabgcMi5kabdMgaPbqab0GaeyyeIuoakiabcYcaSmaalaaabaGae8hXdq3aa0baaSqaaiabdohaZbqaaiabikdaYaaaaOqaaiabd2gaTnaaBaaaleaacqWGPbqAaeqaaaaaaOGaayjkaiaawMcaaiaaxMaacaWLjaWaaeWaaeaacqaIZaWmaiaawIcacaGLPaaaaaa@5A18@

where *s*_*i *_and *s*_*l *_are adjacent areas, and the number of adjacent areas is *m*_*i*_, τs2
 MathType@MTEF@5@5@+=feaafiart1ev1aaatCvAUfKttLearuWrP9MDH5MBPbIqV92AaeXatLxBI9gBaebbnrfifHhDYfgasaacH8akY=wiFfYdH8Gipec8Eeeu0xXdbba9frFj0=OqFfea0dXdd9vqai=hGuQ8kuc9pgc9s8qqaq=dirpe0xb9q8qiLsFr0=vr0=vr0dc8meaabaqaciaacaGaaeqabaqabeGadaaakeaaiiGacqWFepaDdaqhaaWcbaGaem4CamhabaGaeGOmaidaaaaa@3106@ is a spatial variance. The unstructured heterogeneity component was assigned a zero mean Gaussian process with heterogeneity variance τu2
 MathType@MTEF@5@5@+=feaafiart1ev1aaatCvAUfKttLearuWrP9MDH5MBPbIqV92AaeXatLxBI9gBaebbnrfifHhDYfgasaacH8akY=wiFfYdH8Gipec8Eeeu0xXdbba9frFj0=OqFfea0dXdd9vqai=hGuQ8kuc9pgc9s8qqaq=dirpe0xb9q8qiLsFr0=vr0=vr0dc8meaabaqaciaacaGaaeqabaqabeGadaaakeaaiiGacqWFepaDdaqhaaWcbaGaemyDauhabaGaeGOmaidaaaaa@310A@.

The fixed effects (*β*) were assigned diffuse priors. The calendar effect was assigned a second-order random walk priors for flexible smoothing, i.e, *f *(*t*) ~*N *(2*f *(*t *-1) – *f *(*t *- 2), τf2
 MathType@MTEF@5@5@+=feaafiart1ev1aaatCvAUfKttLearuWrP9MDH5MBPbIqV92AaeXatLxBI9gBaebbnrfifHhDYfgasaacH8akY=wiFfYdH8Gipec8Eeeu0xXdbba9frFj0=OqFfea0dXdd9vqai=hGuQ8kuc9pgc9s8qqaq=dirpe0xb9q8qiLsFr0=vr0=vr0dc8meaabaqaciaacaGaaeqabaqabeGadaaakeaaiiGacqWFepaDdaqhaaWcbaGaemOzaygabaGaeGOmaidaaaaa@30EC@), where τf2
 MathType@MTEF@5@5@+=feaafiart1ev1aaatCvAUfKttLearuWrP9MDH5MBPbIqV92AaeXatLxBI9gBaebbnrfifHhDYfgasaacH8akY=wiFfYdH8Gipec8Eeeu0xXdbba9frFj0=OqFfea0dXdd9vqai=hGuQ8kuc9pgc9s8qqaq=dirpe0xb9q8qiLsFr0=vr0=vr0dc8meaabaqaciaacaGaaeqabaqabeGadaaakeaaiiGacqWFepaDdaqhaaWcbaGaemOzaygabaGaeGOmaidaaaaa@30EC@ is a smoothing variance. The variance components (τs2
 MathType@MTEF@5@5@+=feaafiart1ev1aaatCvAUfKttLearuWrP9MDH5MBPbIqV92AaeXatLxBI9gBaebbnrfifHhDYfgasaacH8akY=wiFfYdH8Gipec8Eeeu0xXdbba9frFj0=OqFfea0dXdd9vqai=hGuQ8kuc9pgc9s8qqaq=dirpe0xb9q8qiLsFr0=vr0=vr0dc8meaabaqaciaacaGaaeqabaqabeGadaaakeaaiiGacqWFepaDdaqhaaWcbaGaem4CamhabaGaeGOmaidaaaaa@3106@, τu2
 MathType@MTEF@5@5@+=feaafiart1ev1aaatCvAUfKttLearuWrP9MDH5MBPbIqV92AaeXatLxBI9gBaebbnrfifHhDYfgasaacH8akY=wiFfYdH8Gipec8Eeeu0xXdbba9frFj0=OqFfea0dXdd9vqai=hGuQ8kuc9pgc9s8qqaq=dirpe0xb9q8qiLsFr0=vr0=vr0dc8meaabaqaciaacaGaaeqabaqabeGadaaakeaaiiGacqWFepaDdaqhaaWcbaGaemyDauhabaGaeGOmaidaaaaa@310A@, τf2
 MathType@MTEF@5@5@+=feaafiart1ev1aaatCvAUfKttLearuWrP9MDH5MBPbIqV92AaeXatLxBI9gBaebbnrfifHhDYfgasaacH8akY=wiFfYdH8Gipec8Eeeu0xXdbba9frFj0=OqFfea0dXdd9vqai=hGuQ8kuc9pgc9s8qqaq=dirpe0xb9q8qiLsFr0=vr0=vr0dc8meaabaqaciaacaGaaeqabaqabeGadaaakeaaiiGacqWFepaDdaqhaaWcbaGaemOzaygabaGaeGOmaidaaaaa@30EC@) were assumed to follow an inverse Gamma with parameters 0.001 and 0.001. The Bayesian models were implemented in BayesX [23], using Markov Chain monte carlo (MCMC) simulation techniques. For all models, 50,000 iterations were run with the initial 5,000 discarded and every 15th sample stored to give a final sample of 3,000 for parameter estimation.

## Results

### Population-based results

Table 1 gives the observed number of cases by age group and gender. The person-years at risk, hospitalization and minimum mortality rates are also presented. Incidence rates decreased with increasing age. Infants were the most hospitalised (29.6 per 1,000 person-years), followed by children between the age of 1 to 4 years (17.2 per 1,000 persons). Similarly, most deaths occurred among under-five years old children, with mortality rate of 2.5 per 1000 person-years among infants, and 1.1 per 1,000 person-years among those aged 1–4 years. Rates of hospitalization and in-hospital mortality between boys and girls were similar (11.3 versus 7.8 per 1,000 person-years, and 0.9 versus 0.6 per 1,000 person-years respectively).

**Table 1:** Observed cases of malaria-related hospital admissions and deaths, and person-years at risk by age and sex in Zomba district, Malawi, 2002–2003

Variable		Hospitalized	Dead	Person-years at risk	HR^†^	MMR	CFR^‡ ^(*χ*^2^, *P*-value)
Age	< 1 year	1,189	101	40,188	29.6	2.5	8.5 (15.12, 0.002)
	1–4 years	2,136	133	124,370	17.2	1.1	6.2
	5–9 years	524	55	140,582	3.7	0.4	10.5
	10–14 years	120	13	111,086	1.1	0.1	10.8
Sex	Female	1,683	129	214,626	7.8	0.6	7.7 (0.13, 0.94)
	Male	2,286	173	201,600	11.3	0.9	7.6

Total		3,969	302	416,226	9.5	0.7	7.6

^† ^HR-Hospitalization rate, MMR-minimum mortality rate per 1,000 person-years

^‡ ^CFR-case fatality rate (%)

Results of the Poisson models are given in Table 2. The risk of hospitalization for boys compared to girls was similar, with incidence rate ratio (IRR) equal to 1.02 (95% Confidence Interval (CI): 0.91, 1.14). The risk of hospitalization for infants was higher relative to children aged 10–14 years (IRR: 3.34, 95% CI: 2.80, 3.95). Children aged 5–9 years were at reduced risk compared to children aged 10–14 years (IRR: 0.65, 95% CI: 0.54, 0.78). However, the risk of children between age 1 and 4 years were not different from children aged 10–14 years (IRR: 1.10, 95% CI: 0.93, 1.30). The risk of in-hospital mortality was not different for boys relative to girls (IRR = 1.05, 95% CI: 0.86, 1.29). Infants were at higher risk of dying in hospital relative to children aged 10–14 years (IRR: 3.74, 95% CI: 2.52, 5.39). However, the risk of children aged 1–4 years and those between 5–9 years were comparably similar to those aged between 10 and 14 years (Table 2).

**Table 2:** Fixed and random effects estimates^‡ ^of paediatric malaria-related admissions and deaths in Zomba district, Malawi, 2002–2003

*Variable*		Hospitalization		Mortality	
		IRR	95% CI	IRR^†^	95% CI
Sex	Male	1.02	(0.91, 1.14)	1.05	(0.86, 1.29)
	Female	1.00		1.00	
Age	< 1 year	3.34	(2.80, 3.95)	3.74	(2.52, 5.39)
	1–4 years	1.10	(0.93, 1.30)	1.29	(0.92, 1.87)
	5–9 years	0.65	(0.54, 0.78)	0.73	(0.47, 1.10)
	10–14 years	1.00		1.00	

*Heterogeneity term*					
Unstructured effect	τu2 MathType@MTEF@5@5@+=feaafiart1ev1aaatCvAUfKttLearuWrP9MDH5MBPbIqV92AaeXatLxBI9gBaebbnrfifHhDYfgasaacH8akY=wiFfYdH8Gipec8Eeeu0xXdbba9frFj0=OqFfea0dXdd9vqai=hGuQ8kuc9pgc9s8qqaq=dirpe0xb9q8qiLsFr0=vr0=vr0dc8meaabaqaciaacaGaaeqabaqabeGadaaakeaaiiGacqWFepaDdaqhaaWcbaGaemyDauhabaGaeGOmaidaaaaa@310A@	0.17	(0.001, 0.81)	1.54	(0.002, 9.09)
Structured effect	τs2 MathType@MTEF@5@5@+=feaafiart1ev1aaatCvAUfKttLearuWrP9MDH5MBPbIqV92AaeXatLxBI9gBaebbnrfifHhDYfgasaacH8akY=wiFfYdH8Gipec8Eeeu0xXdbba9frFj0=OqFfea0dXdd9vqai=hGuQ8kuc9pgc9s8qqaq=dirpe0xb9q8qiLsFr0=vr0=vr0dc8meaabaqaciaacaGaaeqabaqabeGadaaakeaaiiGacqWFepaDdaqhaaWcbaGaem4CamhabaGaeGOmaidaaaaa@3106@	7.17	(2.65, 14.11)	21.77	(0.66, 92.41)

^‡ ^Based on spatial Poisson model: Equation 1

^† ^IRR-Incidence rate ratios, Cl-Confidence interval

**Table 3:** Predictors of in-hospital mortality among paediatric patients hospitalised for malaria in Zomba, Malawi, 2002–2003

*Risk factor*		% died (Admissions)	Bivariate model		Multiple spatial model^‡^	
			UOR^†^	95% CI	AOR	95% CI
Age	< 1 year	8.5 (1,189)	1.02	(0.88, 1.18)	1.04	(0.84, 1.28)
	1–4 years	6.2 (2,136)	0.78	(0.68, 0.89)	0.84	(0.74, 0.98)
	5–14 years	10.6 (644)	1.00		1.00	
Sex	Female child	7.7 (1,683)	1.01	(0.92, 1.12)		
	Male	7.6 (2,286)	1.00			
Day	Weekend	7.5 (2,418)	1.01	(0.90, 1.11)		
	Weekday	7.5 (1,492)	1.00			
Season	Wet	6.6 (2,262)	0.88	(0.79, 0.99)	0.79	(0.63, 0.88)
	Dry	8.6 (1,128)	1.00		1.00	
Distance	≥ 5 kms	7.4 (1,938)	0.95	(0.84, 1.03)	0.78	(0.66, 0.94)
	> 5 kms	8.8 (1,999)	1.00		1.00	
Referral	Yes	8.8 (1,895)	1.18	(1.08, 1.35)	2.95	(2.30, 3.75)
	No	6.1 (1,494)	1.00		1.00	
Length of stay	1 day	23.7 (891)	3.71	(2.24, 6.14)	2.56	(1.54, 4.20)
	2 days	2.6 (1,001)	0.32	(0.17, 0.59)	0.24	(0.14, 0.45)
	3 days	2.9 (860)	0.36	(0.19, 0.67)	0.31	(0.17, 0.55)
	4–6 days	2.2 (984)	0.27	(0.14, 0.52)	0.24	(0.13, 0.44)
	7–20 days	7.7 (233)	1.00		1.00	
Treatment	other drugs	7.5 (240)	1.00			
	quinine	7.6 (3,629)	0.98	(0.60, 1.61)		

*Heterogeneity term*^‡^						
Unstructured effect	τu2 MathType@MTEF@5@5@+=feaafiart1ev1aaatCvAUfKttLearuWrP9MDH5MBPbIqV92AaeXatLxBI9gBaebbnrfifHhDYfgasaacH8akY=wiFfYdH8Gipec8Eeeu0xXdbba9frFj0=OqFfea0dXdd9vqai=hGuQ8kuc9pgc9s8qqaq=dirpe0xb9q8qiLsFr0=vr0=vr0dc8meaabaqaciaacaGaaeqabaqabeGadaaakeaaiiGacqWFepaDdaqhaaWcbaGaemyDauhabaGaeGOmaidaaaaa@310A@				2.04	(0.001, 8.75)
Structured effect	τs2 MathType@MTEF@5@5@+=feaafiart1ev1aaatCvAUfKttLearuWrP9MDH5MBPbIqV92AaeXatLxBI9gBaebbnrfifHhDYfgasaacH8akY=wiFfYdH8Gipec8Eeeu0xXdbba9frFj0=OqFfea0dXdd9vqai=hGuQ8kuc9pgc9s8qqaq=dirpe0xb9q8qiLsFr0=vr0=vr0dc8meaabaqaciaacaGaaeqabaqabeGadaaakeaaiiGacqWFepaDdaqhaaWcbaGaem4CamhabaGaeGOmaidaaaaa@3106@				23.74	(0.02, 78.68)

^†^UOR-unadjusted odds ratios, AOR-adjusted odds ratios, CI-confidence intervals

^‡^Based on multiple logistic spatial model: Equation 2

**Figure 1:**
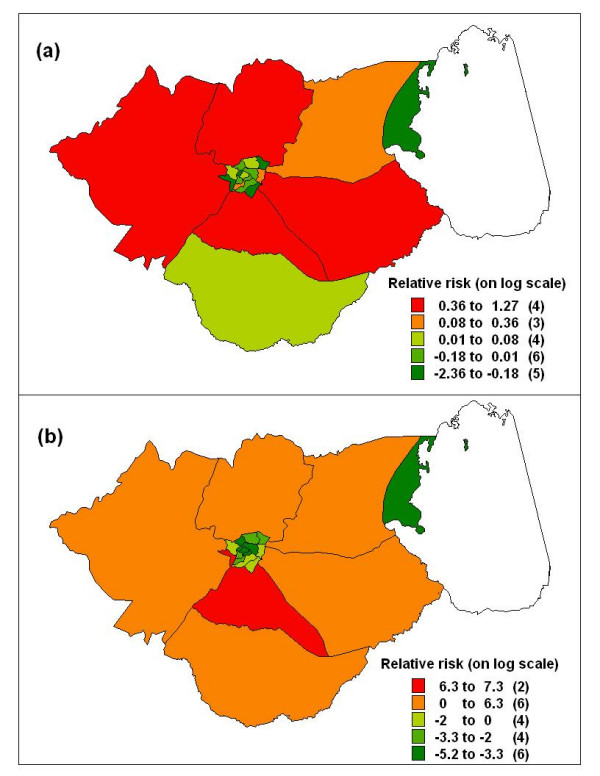
Spatial residual effects of paediatric patients admitted and died of malaria in hospital in Zomba district, Malawi, January 2002 to December 2003: (a) hospitalization, and (b) in-hospital deaths residual effects. The district hospital is located at the centre

Residual spatial effects for hospitalizations and deaths are presented in Figure 1. The top map shows residual spatial effects of hospitalization for malaria, while the bottom plot shows the residual effects of the subsequent in-hospital mortality. Overall in both plots, the risk was higher for those far away from the district centre, and lower for those within the district centre (where the district hospital is located). The unstructured heterogeneity term was small and not significant for both outcomes (Table 2).

### Individual based results

**Figure 2:**
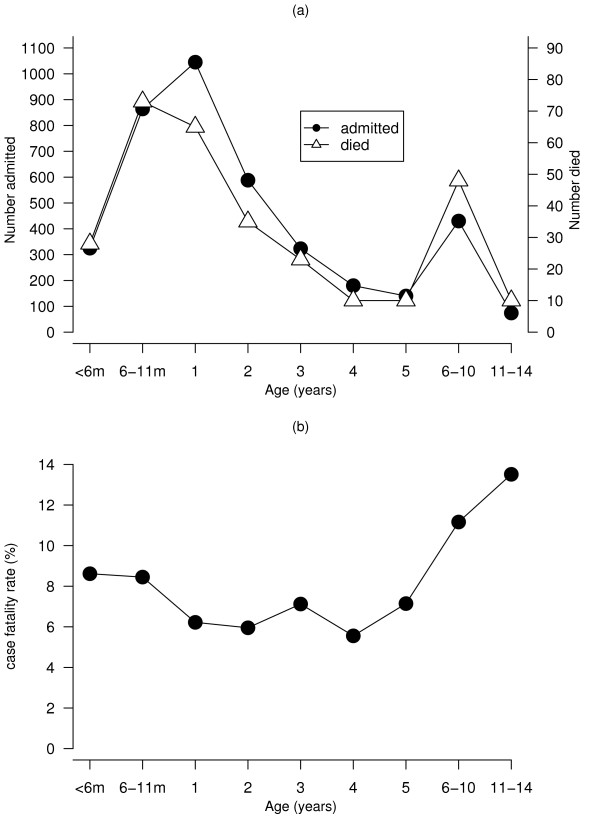
Age distribution of paediatric (a) malaria admissions and death, and (b) case fatality rate at Zomba district hospital, Malawi, January 2002- December 2003

**Figure 3:**
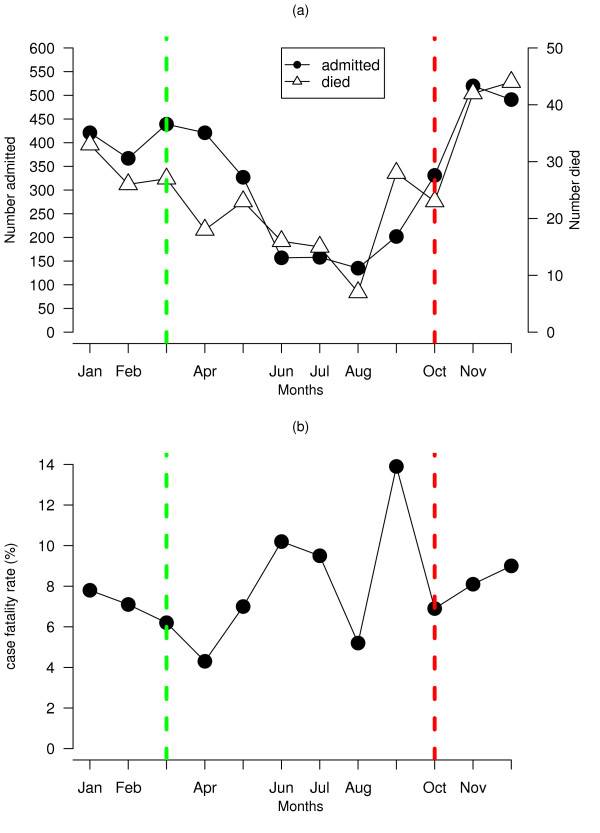
Monthly distribution of paediatric (a) malaria admissions and death, and (b) case fatality rate at Zomba district hospital, Malawi. The vertical lines indicate the start and end of the rainy season (red and green lines respectively)

A total of 302 deaths were registered among 3,969 children hospitalised for malaria, between January 2002 to December 2003, resulting in an overall case fatality rate of 7.6%. Figure 2 shows the number of admission, deaths and case fatality rate by age. The number of admissions and deaths decreased with increasing age, with most deaths (84%) in children under the age of 5 years. The case fatality rate also dropped in the first five years from 8.5% in the age category of < 6 months to 7.1% at age of 5 years, the lowest being at age of 4 years (5.5%) and increased in the last two age groups (Figure 2(b)), but overall the CFR was significantly different (*χ*^2 ^= 15.12, *p <*0.01). Figure 3 shows the monthly distribution of cases hospitalised, died and case-fatality rates. The number of cases were relatively more in the wet season (October-March) compared to the dry season (April-September). However, the pattern of case fatality rate is not quite reflective of the seasonal changes with the highest rates occurring during the dry season, although significant differences were observed (*χ*^2 ^= 24.61, *p *< 0.05).

Table 3 shows the proportion who died in different covariate subgroups. The proportion varied with age, referral status, season, distance from the hospital and length of stay. Quinine was the most administered treatment (94%), compared to other drugs. Boys were more frequently hospitalised than girls (58%), but CFR was not significantly different (*χ*^2 ^= 0.13, *p *= 0.94). The hospital received relatively more patients from a distance of more than 5 kms (52%), with distant patient likely to die in hospital (*χ*^2 ^= 7.2, *p *= 0.054).

The same table gives unadjusted odds ratios (OR) and 95% CI of OR from the bivariate logistic models. Children aged 1–4 years were at reduced risk of dying in hospital relative to children aged 5–14 years (OR = 0.78, 95% CI: 0.68, 0.89). Children referred to the district hospital from networking health facilities were at increased risk of dying in hospital relative to non-referred children (OR: 1.18, 95% CI: 1.08, 1.35). Children hospitalised during the wet season were at reduced risk of dying in hospital relative to those hospitalised during the dry season (OR: 0.88, 95% CI: 0.79, 0.99). Those coming from a distance of within < 5 km were at reduced risk of dying in hospital relative to those coming from a distance of more than 5 km (OR: 0.95, 95% CI: 0.84, 1.03). Length of hospital stay was also associated with the risk of hospital death. Day 1 showed a raised risk relative to 7 or more days in hospital (OR = 3.71, 95% CI: 2.24, 6.14). Day 2, day 3 and day 4–6 recorded reduced risk relative to day 7 or more. No treatment differences were observed between those given quinine as treatment relative to those who received other drugs: OR = 0.98, 95% CI: 0.60, 1.61 (Table 3). Neither sex nor day of admission were significantly associated with hospital deaths.

**Figure 4:**
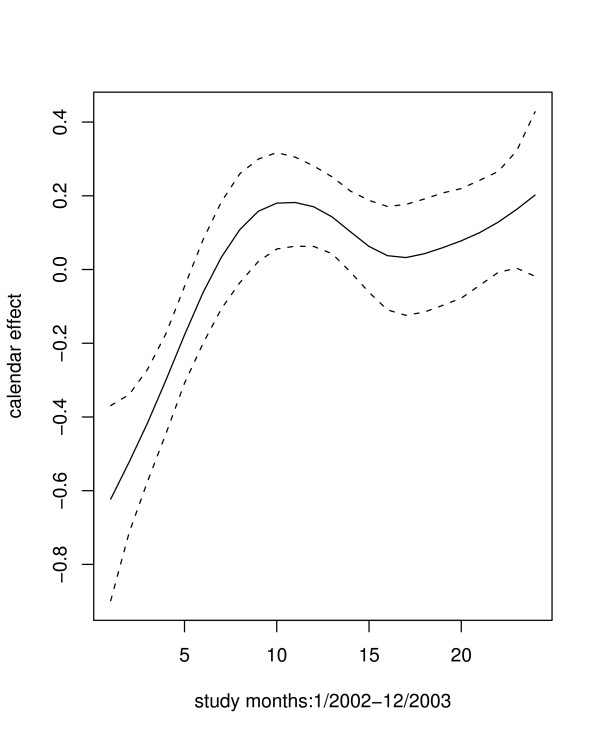
Monthly calendar effect (from January 2002 to December 2003) for the outcome- hospital death with the corresponding 95% confidence lines (dotted line)

**Figure 5:**
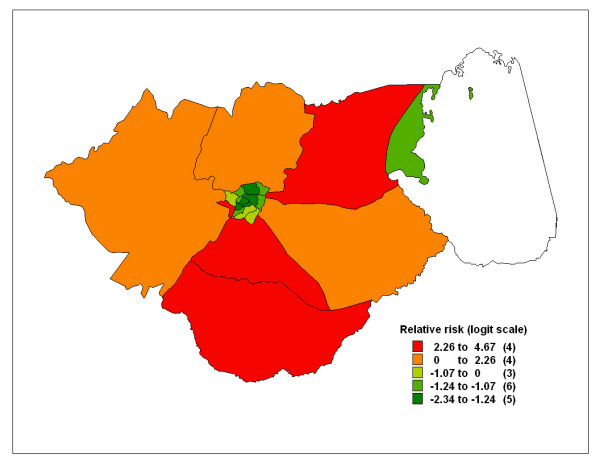
Spatial residuals (i.e., relative odds ratios) of malaria-related hospital deaths based on data from Zomba hospital's paediatric ward, 2002–2003. The estimates were obtained using the multiple spatial logistic model: Equation 2

The multiple spatial logistic regression model included age, season, distance, referral and length of stay as fixed effects. Results were similar to those obtained in the bivariate model (Table 3 – last column). The residual calendar effects for case fatality rate are plotted in Figure 4. The CFR increased with time for the first ten months, decreased up to the 17th month and increased again suggesting seasonal effects, but remained high (positive) for the entire period. Figure 5 shows the residual spatial effects for the case fatality rate. Areas with increased risk (*positive *on logit scale) are relatively far from the general hospital, since the hospital is located at the centre of the district. Areas of lower risk of dying (*negative*) were closer to the district hospital or within the district centre.

Note that residual spatial patterns were similar when compared with those obtained under population based model (Figure 3b), with four areas (Mtiya, Kuntumanji, Mbiza and Chikowi) being the worst, three of which are located in the south. Compared with the residuals in the hospitalization model, two of the areas (Kuntumanji and Mbiza) have high risk of hospitalization, which may suggest that high admission cases translate to high mortality rate in the two areas. The variance components were 2.04 and 23.74 for the unstructured term and structured term respectively.

## Discussion

This study provides evidence of the magnitude and spatial variation of malaria hospitalisation and in-hospital mortality in Zomba, Malawi. In all models, the risk of hospitalisation and in-hospital deaths were highest in areas outside of the district centre (where the district hospital is located). Distant areas with low hospitalisation rates suggest problems of access, which does translate into high mortality rate (Figure 1). However, this is not the case with the southern part of the district. The low risk observed in this area may be explained by availability of relatively better service in the area. The area has three health facilities, two health centres and one rural hospital with resident ambulatory services which may assist promptly with referrals to the district hospital.

The residual spatial heterogeneity (Figures 1 and 5), suggests that unobserved factors not captured by the covariate in the models may contribute to the geographical disparities in the two outcomes. It remains a matter of conjecture to identify such factors, and it is possible that determinants of malaria transmission which are spatially correlated, for example, mosquito breeding sites and habitats may influence the pattern of malaria incidence. This may partly explain the high risk of mortality observed in areas along the Likangala river flowing in the south-east direction, Chikanda township and Lake Chirwa swamps on the eastern side of the district. Moreover, such areas practice irrigated rice agriculture and this system has been associated with increased malaria transmission [24].

Another possible explanation of varied clinical outcomes may be the socio-economic discrepancies in the district. For instance, people from remote or rural areas are relatively poor compared to those at the centre of the district, and these are at increased risk of malaria infection and death because they are not able to pay for effective malaria drugs nor afford transport to a health facility that can treat malaria [25,26]. Rurality is, therefore, one of the factors worth considering in future research. Medical services are often concentrated around trading centres. The further the village is from the trading centre, the more disadvantaged the households are in terms of getting early health care.

Health seeking behaviour plays a critical role in accessing prompt and effective care. Because this was not directly observed, we suggest that this may also explain some of the spatial variation in hospitalisation rates (Figure 1). Home based care or traditional medicines are the first sources of care in most communities [27,28], because of traditional beliefs, difficulties in accessing and unavailability of formal health services [27]. Only when the initial remedies have failed, health centres are the next step [29]. Using a national wide representative survey data, the authors are currently investigating spatial patterns of sources of treatment, including health facility care, for malaria among care-givers of children who had the disease. This may provide some interesting answers on factors affecting health seeking behaviour at community level.

In the 2-year review, the overall case fatality rate of 7.6% was lower than the national rate of 18% [17]. This may be explained by the relatively high altitude location of Zomba, which may lead to low malaria risk. Indeed, recent statistics show that districts at high altitude had relatively low malaria-attributable admissions and mortality compared to other districts in the country [18]. Despite the lower rate, malaria was among the leading causes of death, similar to what was noted in other districts in the country [18], and elsewhere in Africa [10]. Selected reviews on proportional malaria mortality rates in Africa [9,10,12], suggest that Zomba district experienced comparatively low malaria risk between 2002–2003.

The results indicated that risk of malaria hospitalization decreased with increasing age, with those under the age of 1 years at highest risk than subsequent ages (Figure 2 and Tables 1, 2, 3). CFR decreased with age, again infants being the most vulnerable. Overall, under-five children were at high risk, and confirms previous findings in sub-Saharan Africa [30-33]. Children are vulnerable to malaria from about 4 months of age because of waned maternal immunity, and, in highly endemic areas during the peak transmission season, approximately 70% of one-year-olds have malaria parasites in their blood [32,33]. The increase in CFR for those aged 6–14 years, although these are supposed to be protected through acquired immunity, may reflect some aspects of health seeking behaviour, and emphasize the need for prompt and effective management of malaria for all children including those aged over five years even if such cases may not frequently occur in the general population [9,32].

The study showed that patients within 5 km of hospital were less likely to die in hospital than those beyond 5 km, and does reflect the fact that nearness to the hospital improved early access to care [13,34], thus reduced the risk of in-hospital mortality. It was also observed that referral children were at higher risk of dying in hospital, even after adjusting for distance. This seems to suggest that delayed effective treatment (in the process of being transferred to the district hospital) increased the severity of the disease. This could be because most referring health facilities may often be faced with stock-out of effective drugs or may not have prompt access to ambulatory support when needed [20]. Possibilities of interaction between referral and distance might be likely, although this was not significant when included in the model, indicating that referral was independently associated with malaria CFR and not simply due to confounding with distance. This suggests inadequate care being available at primary facilities, regardless of whether they are distant from the hospital or not. It is also possible that referring hospitals are referring the more severe cases which are expected to have a higher case facility rate. Further research is warranted to investigate the timing and availability of pre-referral drugs, and other health facility characteristics that may lead to delayed referral, and suggest ways of improving the referral system in the district. This challenge is similar to other districts in the country [20], and more familiar in most sub-Saharan countries [4].

With regard to the length of hospital stay, it was found that the pattern of hospital deaths was significantly associated with the length of hospital stay. The findings indicated that the sickest patients had a short length of stay terminating in death, with highest risk of dying in hospital the same day of admission. The high CFR on day 1 can be attributed to severe or complicated cases. Indeed in some settings, biomedical care is sought when the condition is near fatal [25,29]. However, as days of stay increased the risk diminished, only to increase again at day 7. This suggests that by and large the care that is provided in the hospital is effective and saves lives, while the increase in risk from day 7 may be a factor of secondary infection although data was not available to investigate this further.

Malaria transmission is more intense in the wet season, yet the results showed that the risk was lower in the wet than the dry season. The likely reason for this is that there were more cases in the wet season (Figure 3), hence the denominator was higher. The huge volume of malaria-related admissions is explained by the increased malaria transmission intensity during the wet season [5,11,12].

These analyses depended on data collected from routine hospital registers. One major shortcoming of using such data is that they only represent those patients who visited the clinics or hospital. As demonstrated in other studies [27,28], and elsewhere in Africa for example in Tanzania [29], most malaria treatments occur outside the formal curative care, and only do so if the illness is perceived to be near fatal. Hence, the true district pattern of hospitalisation and in-hospital mortality may be distorted and underestimated compared to similar tropical setting like Zomba district. For an improved spatial analysis, it would be appropriate to include exact village locations, as opposed to aggregating cases to wards as was done in this study. However, geo-referencing villages would require extra resources as geo-locations are not readily available. Moreover, if such an exercise is undertaken it would be necessary to apply a unique location code that distinguishes locations with similar names which was found to be a challenge in this study.

This study provides evidence that hospital admissions and mortality rates for malaria among Malawian children are high, and that they vary in space. This analysis, accordingly, is the first of its kind and its advantage over other methods is that the impact of location on the two health outcomes was accounted for. These geographical disparities in malaria risk may largely be explained by determinants of malaria transmission, health services availability and accessibility, and health seeking behaviour. Although treatment was not significantly associated with CFR, improved prognosis increased with length of hospital stay indicating that appropriate care when available can save lives. The increased mortality risk for those referred from primary facilities signifies lack of adequate care provided by the primary health care system, but may also be a factor of referring facilities were referring more severe cases, which are expected to have a higher case fatality rate or that patients present later to the hospital if they have been referred. Improved case management at primary facilities by ensuring adequate stocks of effective drugs, combined with home or community interventions, for example, educating the community in management of malaria including training of shopkeepers in the appropriate choice and dose of antimalarial drugs for the treatment of childhood fevers is therefore a high priority [26], and strategies to interrupt malaria transmission through, for instance, indoor residual spraying and insecticide treated nets, are essential to reduce malaria mortality.

## Authors' contributions

LNK conceptualized, analysed and drafted the manuscript. IK and BLS participated in the conception, and critical review of the manuscript.
